# Elongation Factor P Interactions with the Ribosome Are Independent of Pausing

**DOI:** 10.1128/mBio.01056-17

**Published:** 2017-08-01

**Authors:** Rodney Tollerson, Anne Witzky, Michael Ibba

**Affiliations:** a Department of Microbiology, The Ohio State University, Columbus, Ohio, USA; b Department of Molecular Genetics, The Ohio State University, Columbus, Ohio, USA; c Center for RNA Biology, The Ohio State University, Columbus, Ohio, USA

**Keywords:** elongation factor P, pausing, translation

## Abstract

Bacterial elongation factor P (EF-P) plays a pivotal role in the translation of polyproline motifs. To stimulate peptide bond formation, EF-P must enter the ribosome via an empty E-site. Using fluorescence-based single-molecule tracking, Mohapatra et al. (S. Mohapatra, H. Choi, X. Ge, S. Sanyal, and J. C. Weisshaar, mBio 8:e00300-17, 2017, https://doi.org/10.1128/mBio.00300-17) monitored the cellular distribution of EF-P and quantified the frequency of association between EF-P and the ribosome under various conditions. Findings from the study showed that EF-P has a localization pattern that is strikingly similar to that of ribosomes. Intriguingly, EF-P was seen to bind ribosomes more frequently than the estimated number of pausing events, indicating that E-site vacancies occur even when ribosomes are not paused. The study provides new insights into the mechanism of EF-P-dependent peptide bond formation and the intricacies of translation elongation.

## COMMENTARY

Of the proteogenic amino acids, proline plays a unique role, providing critical kink turns in proteins and increasing peptide stability (1). For example, in *Mycobacterium tuberculosis*, nearly 10% of the coding capacity is spent on translating proteins containing PE and PPE motifs (2). A less extreme but equally remarkable case is valyl-tRNA synthetase, which contains a universally conserved triprolyl motif required for substrate specificity (3). Due to its unique pyrrolidine ring structure, proline is both a poor peptide bond donor and acceptor, forming peptide bonds with puromycin from 10 to 1,000 times slower than other amino acids (4). Consequently, stretches of prolines can cause ribosome pausing and translational arrest. Unresolved ribosome pausing at polyproline motifs in bacteria can lead to loss of motility, virulence, and cell viability (4, 5). Bacteria use elongation factor P (EF-P) to alleviate ribosome pausing on polyproline motifs. *Escherichia coli* ribosome profiling data have shown that in the absence of EF-P, nearly half of PPX (where X is any third amino acid) motifs have a >10-fold-higher rate of ribosome occupancy than when EF-P is absent, which is indicative of strong pausing at these motifs (6).

EF-P must be posttranslationally modified at a conserved residue to perform its function. Genes required for modification are highly pleiotropic, and knockout mutants mimic phenotypes displayed in *efp* deletion backgrounds. Though EF-P and its homologues are conserved throughout all domains of life, the structures of the posttranslational modification (PTM) are not. For example, the *E. coli* and *Bacillus subtilis* EF-Ps are modified by the linear (*R*)-β-lysine and 5-aminopentanol, respectively, while the *Pseudomonas aeruginosa* and *Neisseria menengitidis* EF-Ps are modified by a cyclic rhamnose moiety. The exact mechanism of EF-P-mediated ribosome rescue is currently unknown, but it is speculated that when modified, EF-P increases the stability of the P-site tRNA and proline within the ribosomal peptidyl transfer center (PTC), possibly changing the orientation of proline to be more amenable for peptide bond formation (7). Biochemical work suggests that the *E. coli* EF-P posttranslational modification, (*R*)-β-lysine, significantly increases the association constant between EF-P and the ribosome, though the mechanism of this phenomenon is unknown (4). Curiously, using the *Thermus thermophilus* crystal structure of EF-P bound to the 70S ribosome as a guide, rhamnose-modified EF-P seems to be unable to reach into the PTC in the same way that linear modifications would (8). This further confounds our understanding of the role of EF-P PTM, since structurally diverse modifications seem to play similar roles in facilitating peptide bond synthesis.

To further investigate the role of EF-P and its PTM in peptide bond formation, Mohapatra et al. used fluorescence microscopy to identify and quantify the interactions between EF-P and the ribosome through changes in spatial distribution and diffusion rates of the two factors (9). The authors of the study aimed to address a critical question in the field: does EF-P selectively bind ribosomes paused at polyproline motifs via an empty E-site, or does this interaction occur randomly? Since EF-P accelerates peptide bond synthesis within paused ribosomes, one possibility is that ribosome pausing is a prerequisite for EF-P rescue (3). Previous studies have presented evidence that tRNA translocation during elongation is a modular event, making E-site vacancy rare outside spontaneous diffusion (10). In this case, only paused ribosomes would have empty E-sites, and EF-P entry via the E-site would be limited to the population of paused ribosomes.

To determine the axial distribution and diffusion rate of EF-P and ribosomes in *E. coli*, Mohapatra et al. utilized mEos2-tagged EF-P and S2 ribosomal proteins. The utility of the mEos2 tag was the authors’ ability to specifically follow one to two individually tagged molecules at a time. By shifting the wavelength emitted through weak laser excitation, the investigators were able to accurately identify the location of specific molecules at 2-ms intervals. The data generated in their study provided evidence that EF-P and the ribosome share a similar distinctive three-peaked distribution, near the nucleoid and at the cell poles, within an *E. coli* cell under normal conditions. When the ribosomal spatial distribution is perturbed by the addition of antibiotics, EF-P distribution is similarly affected. With addition of the translation-arresting agent chloramphenicol, ribosomes and EF-P had a more nucleoid distribution. In contrast, treatment with a transcription inhibitor, rifampin, caused both factors to be more evenly distributed throughout the cell. Though distribution of EF-P very strikingly mimics that of the ribosome, the spatial organizations are not identical. Axial distribution of EF-P is not as segregated as that of the ribosome, implying that they are not in constant interaction.

As mentioned above, EF-P posttranslational modification has been seen to dramatically increase association between EF-P and the ribosome. To determine the effect of EF-P modification on its axial distribution, mEos2-tagged EF-P^K34A^, which cannot be modified, was expressed from a plasmid in *E. coli* harboring the chromosomal copy of wild-type EF-P to prevent defects associated with only nonfunctional EF-P. Axial distribution of EF-P^K34A^ displayed a pattern similar to that of wild-type EF-P and ribosomes when *E. coli* cells were treated with rifampin; there was no obvious spatial organization, similar to that of a simulated homogeneous distribution. These findings correlate with the *in vitro* biochemical data, providing evidence that the K34A mutant has a major decrease in affinity for the ribosome (4). However, it is difficult to assess the impact that the continued presence of the wild-type copy of EF-P would have had in these experiments.

To identify the fraction of EF-P that interacts with the ribosome, single-step diffusion dynamics were determined for EF-P under a variety of conditions. Quantifications of EF-P–ribosome interactions were made by comparing the rate of diffusion of EF-P to that of the ribosome and determining the best fit in a two-state model. These states are “slow,” in which EF-P slows diffusion by interacting with another factor, in this case the ribosome, and “fast,” in which EF-P is not bound to ribosomes and is freely diffusing. By comparing the fraction of “slow” EF-P molecules to those which were “fast,” the Mohapatra group determined the fraction of ribosomes interacting with EF-P. Under normal conditions, the authors estimated that EF-P interacts with anywhere from 25% to 100% of translating ribosomes. This is in stark contrast with their findings that about 0.05% of the active *E. coli* translatome contains a PPX motif, with about 280 motifs present per cell. Therefore, EF-P must “interrogate” ribosomes much more frequently than would be possible if paused ribosomes were the only target.

Curiously, the percentage of total EF-P interacting with ribosomes increases from 30% to 45% when chloramphenicol is added to the cells, possibly due to an increased number of empty E-sites, and in this case even the “fast” EF-P molecules may be interacting transiently with ribosomes (inferred from a decrease in the “fast” mean diffusion coefficient, from 4.3 µm^2^/s to 1.2 µm^2^/s). EF-P^K34A^ also conformed to a two-state model, but both fast and slow diffusion coefficients were strongly divergent from that of EF-P interacting with a ribosome, implying that EF-P^K34A^ may never interact with translating ribosomes in a directed manner.

Through investigation of the overall interaction cycle between EF-P and the ribosome (about 23 ms), the calculated rate constant of EF-P binding to ribosomes was seen to be around 100 times lower than if binding was purely based on diffusion rates, highlighting the intricacies of EF-P interactions with the ribosome. Interestingly, the amount of time in which EF-P is bound to the ribosome is independent of whether translation elongation occurs; in effect, EF-P is randomly interacting with ribosomes independent of pausing. The time between binding events is shorter if translation is arrested, possibly due to the greater availability of ribosomes with empty E-sites.

**FIG 1:**
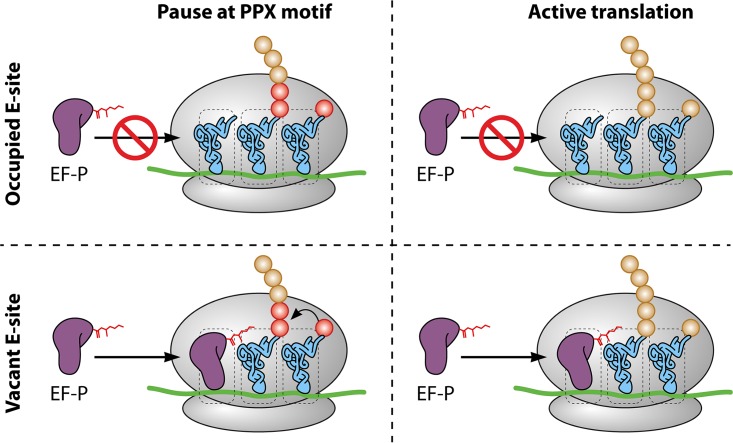
Constant interrogation of ribosomes by posttranslationally modified EF-P. (Top row) Independent of the pausing at an EF-P-dependent (PPX) motif (red circles), EF-P is unable to enter the ribosome via the E-site if the site is occupied by tRNA. (Bottom row) When the E-site is vacant, modified EF-P is able to bind to the ribosome. When the ribosome is paused at a PPX motif, the binding event stimulates peptide bond formation. EF-P binding to ribosomes with empty E-sites due to tRNA diffusion from actively translating ribosomes does not stimulate peptide bond formation.

Mechanistically, EF-P function is intrinsically tied to the availability of vacant ribosomal E-sites. The Mohapatra et al. study showed that not only does EF-P localize with ribosomes in the cell, but also that it is constantly interrogating ribosomes for available E-sites. When successful, the time EF-P spends in the E-site is independent of translational pausing. This paints a picture where EF-P is acting on any open E-site, but only ribosomes pausing at specific motifs benefit from this event (Fig. 1). Lacking a specific sequence which recruits EF-P may be advantageous because of the expansive variety of EF-P-dependent pause motifs and the effect that distal sequences can have on pausing (11). This mechanism can only be viable if E-sites are empty at a much greater frequency than previously expected. The original mechanism of immediate occupation of the vacant E-site by P-site tRNA would not allow for EF-P interrogations occurring with anywhere from 25% to 100% of translating ribosomes. Frequent E-site vacancy also provides evidence that translation elongation is a dynamic process in which translocation of P-site tRNA is not required for E-site tRNA diffusion. The Mohapatra et al. study findings advance not only the field of EF-P-dependent translation but also that of general bacterial translation, where a greater understanding of the elongation cycle is paramount.

## References

[1] Pavlov MY, Watts RE, Tan Z, Cornish VW, Ehrenberg M, Forster AC 2009 Slow peptide bond formation by proline and other N-alkylamino acids in translation. Proc Natl Acad Sci U S A 106:50–54. doi:10.1073/pnas.0809211106. 19104062 PMC2629218

[2] Mukhopadhyay S, Balaji KN 2011 The PE and PPE proteins of Mycobacterium tuberculosis. Tuberculosis 91:441–447. doi:10.1016/j.tube.2011.04.004. 21527209

[3] Lassak J, Wilson DN, Jung K 2016 Stall no more at polyproline stretches with the translation elongation factors EF-P and IF-5A. Mol Microbiol 99:219–235. doi:10.1111/mmi.13233. 26416626

[4] Doerfel LK, Rodnina MV 2013 Elongation factor P: function and effects on bacterial fitness. Biopolymers 99:837–845. doi:10.1002/bip.22341. 23828669

[5] Yanagisawa T, Takahashi H, Suzuki T, Masuda A, Dohmae N, Yokoyama S 2016 Neisseria meningitidis translation elongation factor P and its active-site arginine residue are essential for cell viability. PLoS One 11:e0147907. doi:10.1371/journal.pone.0147907. 26840407 PMC4739656

[6] Mohammad F, Woolstenhulme CJ, Green R, Buskirk AR 2016 Clarifying the translational pausing landscape in bacteria by ribosome profiling. Cell Rep 14:686–694. doi:10.1016/j.celrep.2015.12.073. 26776510 PMC4835026

[7] Blaha G, Stanley RE, Steitz TA 2009 Formation of the first peptide bond: the structure of EF-P bound to the 70S ribosome. Science 325:966–970. doi:10.1126/science.1175800. 19696344 PMC3296453

[8] Lassak J, Keilhauer EC, Fürst M, Wuichet K, Gödeke J, Starosta AL, Chen JM, Søgaard-Andersen L, Rohr J, Wilson DN, Häussler S, Mann M, Jung K 2015 Arginine-rhamnosylation as new strategy to activate translation elongation factor P. Nat Chem Biol 11:266–270. doi:10.1038/nchembio.1751. 25686373 PMC4451828

[9] Mohapatra S, Choi H, Ge X, Sanyal S, Weisshaar JC 2017 Spatial distribution and ribosome-binding dynamics of EF-P in live *Escherichia coli*. mBio 8:e00300-17. doi:10.1128/mBio.00300-17. 28588135 PMC5461404

[10] Chen C, Stevens B, Kaur J, Smilansky Z, Cooperman BS, Goldman YE 2011 Allosteric vs. spontaneous exit-site (E-site) tRNA dissociation early in protein synthesis. Proc Natl Acad Sci U S A 108:16980–16985. doi:10.1073/pnas.1106999108. 21969541 PMC3193197

[11] Elgamal S, Katz A, Hersch SJ, Newsom D, White P, Navarre WW, Ibba M 2014 EF-P dependent pauses integrate proximal and distal signals during translation. PLoS Genet 10:e1004553. doi:10.1371/journal.pgen.1004553. 25144653 PMC4140641

